# Financial and Psychological Risk Attitudes Associated with Two Single Nucleotide Polymorphisms in the Nicotine Receptor (*CHRNA4*) Gene

**DOI:** 10.1371/journal.pone.0006704

**Published:** 2009-08-20

**Authors:** Brian E. Roe, Michael R. Tilley, Howard H. Gu, David Q. Beversdorf, Wolfgang Sadee, Timothy C. Haab, Audrey C. Papp

**Affiliations:** 1 Department of Agricultural, Environmental and Development Economics, Ohio State University, Columbus, Ohio, United States of America; 2 Division of Science and Math, Central Methodist University, Fayette, Missouri, United States of America; 3 Departments of Radiology, Neurology and Psychology and the Thompson Center for Autism and Neurodevelopmental Disorders, University of Missouri-Columbia, Columbia, Missouri, United States of America; 4 Department of Pharmacology and Psychiatry, Ohio State University, Columbus, Ohio, United States of America; University of Wuerzburg, Germany

## Abstract

With recent advances in understanding of the neuroscience of risk taking, attention is now turning to genetic factors that may contribute to individual heterogeneity in risk attitudes. In this paper we test for genetic associations with risk attitude measures derived from both the psychology and economics literature. To develop a long-term prospective study, we first evaluate both types of risk attitudes and find that the economic and psychological measures are poorly correlated, suggesting that different genetic factors may underlie human response to risk faced in different behavioral domains. We then examine polymorphisms in a spectrum of candidate genes that affect neurotransmitter systems influencing dopamine regulation or are thought to be associated with risk attitudes or impulsive disorders. Analysis of the genotyping data identified two single nucleotide polymorphisms (SNPs) in the gene encoding the alpha 4 nicotine receptor (*CHRNA4*, rs4603829 and rs4522666) that are significantly associated with harm avoidance, a risk attitude measurement drawn from the psychology literature. Novelty seeking, another risk attitude measure from the psychology literature, is associated with several *COMT* (catechol-*O*-methyl transferase) SNPs while economic risk attitude measures are associated with several *VMAT2* (vesicular monoamine transporter) SNPs, but the significance of these associations did not withstand statistical adjustment for multiple testing and requires larger cohorts. These exploratory results provide a starting point for understanding the genetic basis of risk attitudes by considering the range of methods available for measuring risk attitudes and by searching beyond the traditional direct focus on dopamine and serotonin receptor and transporter genes.

## Introduction

Risk attitudes have been correlated to a broad array of financial, employment, health, safety and social decisions made by humans, including financial investments, insurance coverage, smoking, drinking, sport participation, migration and self-employment status [1],[2],[3]. Risk attitudes have also been hypothesized as susceptibility factors for pathological gambling [4], [5], [6], anxiety and mood disorders [7], [8], [9] and as a susceptibility factor for impulse control disorders (ICD) among Parkinson's patients on dopamine therapies [10]. Risk attitudes have also been correlated to neurological responses during imaging studies featuring decision making tasks [11], [12] and to the volume of key brain regions [13], [14].

Given the emerging interest in risk attitude measures at the nexus of economics, psychology and neurology, attention is now turning to possible genetic factors that contribute to individual heterogeneity in risk attitudes. Such an interest is buttressed by twin studies that have estimated the heritability of risk attitudes near 20% among a Swedish sample [15] and near 60% among a Chinese sample [16], and by twin studies that estimate the heritability of pathological gambling, which has been estimated near 60% in a U.S. sample [17]. Several studies have attempted to associate risk attitudes with particular genotypes. However these studies differ in terms of how risk attitude is defined and measured and in terms of types of genetic variation investigated.

The risk attitude measures used in genetics studies emerge from two different bodies of literature. Risk attitudes such as harm avoidance (HA) and novelty seeking (NS) are the most commonly explored risk attitudes in behavioral genetics studies. These measures originate in the psychology literature (Temperament and Character Inventory, [18]) and are used broadly by behavioral neuroscientists and geneticists. These measures are derived from a subject's self evaluation of the goodness of fit between various statements concerning a wide range of behaviors (e.g., “I jump into things without thinking…”) and his or her own personality and behavior. NS and HA were originally hypothesized to be driven by variation in the dopamine and serotonin systems, respectively [19]. Several studies have shown associations in NS and HA with polymorphisms thought to affect dopamine and serotonin receptors and transporters, including *DRD2*, *DRD4*, *DRD5* and *DAT* [20], [21], [22] and *SERT* [23], [24]. NS or HA also have been associated with polymorphisms thought to impact dopamine and serotonin synthesis or metabolism, e.g., catechol-O-methyl transferase (*COMT*, [25]) and tryptophan hydroxylase-2 gene (*TPH2* [26]). Other research has pointed to genetic associations beyond dopamine and serotonin, such as brain-derived neurotrophic factor (*BDNF*, [27]).

The second type of risk attitude measurement comes from the economics literature. Economists commonly measure risk attitudes by recording subjects' decisions among competing financial gambles or investment opportunities. Economic risk attitudes often show a limited correlation to psychologically based measurements of risk attitude [28], suggesting that risk attitudes may be domain specific [29] and not captured precisely by more general risk attitude measures such as HA and NS. Risk attitudes derived from financial choice tasks have also been associated with genetic variation, though less work has focused on this class of risk attitude measurements. For example, Roussos et al. [30] associated risk tendencies measured during the Iowa Gambling Task with a *COMT* polymorphism; Kuhnen and Chiao [31] associated risky investment behavior observed during an investment game with polymorphisms in *SERT* and *DRD4*; and Dreber et al. [32] associated financial risk taking with a *DRD4* polymorphism.

In this paper we test for genetic associations with risk attitude measures derived from both the psychology and economics literature. In our sample we find that risk attitude measures from these different literatures are not strongly correlated. Given the range of genes implicated in past genetics studies, we search for associations across polymorphisms in a spectrum of candidate genes that affect neurotransmitter systems influencing dopamine regulation or in genes thought to be associated with risk attitudes or impulsive disorders.

We find two single nucleotide polymorphisms (SNPs) in the gene encoding the alpha 4 nicotine receptor (*CHRNA4*, rs4603829 and rs4522666) are associated with harm avoidance in our sample of college normals. Both associations are significant after the false discovery rate adjustment is implemented to account for multiple testing. The rs4603829 SNP, which is located in the 3′ region, has not been associated previously with a phenotype. Given the exploratory nature of our study and the limited size of our sample (n = 67) we also catalogue 22 associations that are significant in the absence of statistical adjustment for multiple testing. These 22 associations include involve risk attitude measures derived from both the economics and psychology literature and SNPs affecting several genes. In particular novelty seeking is associated with several *COMT* SNPs while economic risk attitudes are associated with several *VMAT* SNPs. These findings suggest an improved understanding of the genetic basis of risk attitudes must consider the breadth of methods available for measuring risk attitudes and look beyond the traditional direct focus on dopamine and serotonin receptor and transporter genes.

## Materials and Methods

### Subjects

The study involved 67 subjects (29 female, 8 non-white, mean age = 20.6 years, standard deviation of age = 3.2 years) and was approved by the local Biomedical Internal Review Board. Subjects were recruited via e-mail from interested university students and local community members. Exclusion criteria included left-handedness, color blindness, pregnancy, brain aneurysm, cognitive impairment, and age less than 18; many criteria were included so that all subjects might be eligible for subsequent brain imaging experiments not reported here. Self-reported health histories were collected; 12 subjects reported ever being diagnosed with depression, bi-polar disorder or another psychiatric disorder. All subjects described themselves as either a non-user of tobacco (n = 57) or an occasional user of tobacco (not daily, n = 9). All subjects completed the preferred gambles task while 65 subjects completed the personality traits inventory. The entire experiment took approximately 1.5 hours and the average subject payment was $50; actual payment ranged from $34 to $131 and depended upon choices made during the preferred gambles task and the roll of a die.

### Risk Attitude Tasks

#### A Preferred Gambles Task from the Economics Literature

This task assesses the tendency for individuals to seek out risky rather than conservative financial opportunities, which has been show to predict pathological gambling behavior [33] and to be stable over periods of at least 17 months [34], suggesting the task is measuring an individual trait rather than a temporary state regarding financial risk tolerance. In this task, which is adapted from the economics literature [35], respondents are presented with a sequence of choices. Each choice is between two gambles: a safe gamble (e.g., Option A: a 1-in-10 chance of gaining $15 and a 9-in-10 chance of gaining $12) and a risky gamble (e.g., Option B: a 1-in-10 chance of gaining $29 and a 9-in-10 chance of gaining $0.75). Before each decision, respondents are informed of the difference in the expected values of the two gambles (for the previous example, the respondent is informed: “If both gambles were played 100 times Option A would usually gain $8.73 more than Option B). After reading the information about the pair of gambles, respondents must mark one of the two gambles as preferred on the paper form.

Each respondent is presented with 36 gamble pairs in 4 blocks of 9. Each choice features one safe gamble and one risky gamble, though the neutral language ‘Option A’ and ‘Option B’ is used in place of ‘safe’ and ‘risky’. Within each block the dollar values for the safe and risky gamble payouts are fixed while probability of winning the higher dollar value is increased by 0.1 with each additional choice. For example, continuing the example above, the second choice in the block would be feature an Option A with a 2-in-10 chance of gaining $15 and an 8-in-10 chance of gaining $12.

In Block 2 the gamble pairs are similar to the gamble pairs in Block 1 in terms of the magnitude of rewards at stake and in terms of the probabilities of receiving high versus low payments. However, in Block 2 each gamble is now described as a loss rather than a gain. Specifically, respondents are informed that they begin each decision in Block 2 with $30 and must choose between two gambles where all outcomes will result in losing some portion of that $30 endowment. For example, for the first choice in this block, Option A is a 1-in-10 chance of losing $15 and a 9-in-10 chance of losing $18, while Option B is a 1-in-10 chance of losing $1 and a 9-in-10 chance of losing $29.25.

Blocks 3 and 4 mirror Blocks 1 and 2 in all aspects expect the absolute dollar values, which are increased to $50 and $40 for Option A and $98 and $2.50 for Option B. The gamble pairs in Block 3 are described as gains while the gamble pairs in Block 4 are described as losses from a base endowment of $100.

After all subjects chose a preferred gamble from all 9 pairs in each of the 4 blocks, the experimental moderator collected the completed forms and then publicly rolled dice to determine which of the 18 small-magnitude gamble pairs (Blocks 1 and 2) would be chosen as the basis for payment. That is, only 1 of the 18 gamble pairs was played for real cash; the subjects were aware of this prior to choosing and told to treat all choices with equal seriousness. The experimental moderator rolled another 10-sided die to determine whether each subject received the high or low cash payment from their preferred gamble. Finally 1 in 30 subjects was randomly chosen to receive the large-stakes version of their chosen gamble in place of the small rewards version. Subjects were briefed on the nature of this compensation scheme prior to their selection of preferred gambles.

Two measures result from this task. The first is the % Safe Gambles among Gains (SGG) measure, which is the percent of safe gambles chosen from all gamble pairs described as gains (Blocks 1 and 3). The second is the % Safe Gambles among Large Stakes (SGL) measure, which is the percent of safe gambles chosen from all large magnitude gambles (Blocks 3 and 4). Past research in economics suggests that subjects act more conservatively when gambles are framed as gains rather than losses [36] and act more conservatively when the absolute rewards involved in gambles increase [35].

#### Personality Traits Novelty Seeking and Harm Avoidance from the Psychology Literature

Two temperament/personality phenotypes are measured: novelty seeking (NS) and harm avoidance (HA), both of which are included in the Temperament and Character Inventory [18]. NS captures a subject's tendency toward exploratory activity and exhilaration in response to novel stimuli, while HA captures the intensity of a subject's response to aversive stimuli and eagerness to avoid such stimuli. Numeric scores for NS and HA are based on subjects' ratings of 35 statements for NS and 39 statements for HA where the statements are drawn from the International Personality Inventory Pool [37], a public-domain instrument shown to correlate to major personality inventories including the Temperament and Character Inventory [38]. The scoring procedure is described elsewhere [37]. All NS and HA scores are expressed as a percent of the maximum potential score. Both NS and HA are designed as trait measures, as instructions direct individuals to assess the statements in relation to how the subject generally see himself/herself, though research suggests that HA may be influenced by the onset of major psychological disorders such as depression [39].

HA has been associated with neurological response to hypothetical financial risk games presented in fMRI experiments [11], [40] while NS and HA have been associated with pathological gambling behavior [4], [5], [6], [41].

Table 1 presents summary statistics for each of the risk attitude measures in addition to a Spearman Rank correlation matrix. The p-values for the correlations are adjusted for multiple testing using the Sidak adjustment. Pearson correlations yield similar qualitative and quantitative results and are not reported. Tests (both standard *t*-tests and the non-parametric Kruskal-Wallis tests) by gender and race reveal no statistically significant differences across these sub-groups for any risk attitude measure. Risk attitudes derived from the same disciplinary field display significant within-field correlation (HS and NA, SGG and SGL), while correlations of risk attitude measures from different disciplines are uncorrelated and of similar magnitude as previously documented [28].

**Table 1:** Spearman Rank Correlation Coefficients Among Tasks.

	NS	HA	SGL	SGG	Summary Statistics
Novelty Seeking	1.000				0.437
(NS)	–	–	–	–	0.013
	64				(0.179, 0.736)
Harm Avoidance	-0.411	1.000			0.426
(HA)	0.004	–	–	–	0.018
	64	64			(0.135, 0.763)
% Safe Gambles – Large Stakes	-0.063	0.006	1.000		0.387
(SGL)	0.997	1.000	–	–	0.020
	64	64	66		(0, 0.667)
% Safe Gambles – Gains	-0.066	-0.179	0.886	1.000	0.403
(SGG)	0.996	0.642	<0.001	–	0.019
	64	64	66	66	(0.056, 0.778)

Each cell in the first 4 columns of numbers lists the Spearman rank correlation coefficient, Sidak adjusted *p*-value and number of observations, respectively. Each summary statistic cell lists mean, standard error, minimum and maximum observation, respectively.

### Genotypes

Genotyping focused on 98 polymorphisms in genes thought to affect catecholamine function, or previously implicated in impulse control issues or associated with risk attitudes. The SNP choice was calculated with Applied Biosystems SNP Browser 3.0 software using the haplotype tagging method (r^2^ = .95). Priority was given to SNPs located in transcribed RNA (see Table 2). Putative variable number tandem repeats (VNTR's) in estrogen receptor 1, dopamine transporter and monoamine oxidase were also analyzed ([42], [43], [44], see Table 3). Genomic DNA was isolated from whole blood using the Flexigene kit (Qiagen). After DNA isolation, DNA concentration and purity was determined by calculating the A_260_/A_280_ ratio. The subject's genotype was determined by the pharmacogenomics core facility at The Ohio State University, Department of Pharmacology using SNPlex (Applied Biosystems, Forster City, CA) or fluorescent PCR. Polymorphisms were checked for Hardy-Weinberg equilibrium (p<0.05) and a minimum minor allele frequency of 5% using Helix Tree (Golden Helix, Bozeman, Montana). A total of 81 SNPs (denoted in Table 2) and all three VNTRs (Table 3) passed these tests and were used in the association analysis.

**Table 2:** List of SNPs Analyzed.

	Gene	SNP
*CHRNA4*	Cholinergic receptor, nicotinic, alpha 4	rs755203, rs1044393, rs1044397*, rs2093107, rs2236196, rs2273502, rs4522666, rs4603829
*COMT*	Catechol-*O*-methyl transferase	rs4633, rs4680*, rs4818, rs165656, rs165722, rs174699, rs4646312, rs740603*
*DAO*	D-amino acid oxidase	rs2070588, rs3741775, rs3825251, rs3918347, rs7980427
*DAT*	Dopamine transporter	rs6347*, rs6350*, rs27072, rs37022, rs40184, rs1042098, rs2937639, rs464049
*DISC1*	Disrupted-in-Schizophrenia 1	rs823163, rs913730, rs1000731, rs3738401
*DRD2*	Dopamine receptor D_2_	rs6277, rs6279, rs1079595, rs1124493, rs1125394, rs1800497*, rs1984739, rs2075654, rs2242446, rs4648318, rs6589377, rs7117915, rs7125415, rs11214608, rs12364283
*DRD3*	Dopamine receptor D_3_	rs6280, rs226082, rs963468, rs2134655
*ESRa*	Estrogen receptor alpha	rs827421, rs988328, rs1801132, rs2228480, rs3798577
*HTR2A*	Serotonin receptor subtype 2_A_	rs6313, rs2070039, rs2246127
*MAOA*	Monoamine oxidase A	rs6323*, rs909525*, rs979605*, rs979606*, rs1801291*, rs3027407*
*MAOB*	Monoamine oxidase B	rs17462, rs3027452*, rs7879356*
*NET*	Norepinephrine transporter	rs3081, rs5569, rs15534, rs36017, rs40434, rs998424, rs2242446, rs2242447, rs2279805
*SERT*	Serotonin transporter	r140701, r1872924, r2020934, r3783594, r3794808
*TH*	Tyrosine hydroxylase	rs6356, rs6357, rs2070762, rs4074905
*TPH2*	Tryptophan hydroxylase 2	rs4290270, rs7305115
*VMAT2*	Vesicular Monoamine Transporter 2	rs14240, rs36339, rs363333, rs363343, rs929493, rs1860404

* SNPs that fail screening criteria for HE equilibrium and sufficient minor allele frequency.

**Table 3:** List of Variable Number of Tandem Repeats Analyzed.

	Gene	Repeat Groupings
*DAT*	Dopamine transporter	9, 10 or more
*ESRa*	Estrogen receptor alpha	18 or fewer, 19 or more
*MAOA*	Monoamine oxidase A	3, 4

### Statistical Methods

The genetic association tests used are F-tests of the difference in the distribution of the risk attitude measure across subjects in different genotype categories; we implement both a dominant and a recessive model for each polymorphism and phenotype considered. The p-value is corrected for multiple testing using the false discovery rate (FDR) p-value [45]. FDR is an alternative that controls the number of false positives that is equivalent to the Bonferroni method when there are no truly significant results; otherwise it is less conservative and therefore a more powerful test [46].

## Results

### Harm Avoidance and CHRNA4

Two *CHRNA4* SNPs (rs4603829 and rs4522666) showed a significant association with HA score (F _1, 62_ = 14.34, p = 0.029 and F _1, 57_ = 11.95, p = 0.042, respectively after FDR correction) using a recessive genetic association model. Both of these SNPs are located in the 3′ gene region and thus do not affect the amino acid sequence of *CHRNA4*, but could affect alpha4 signaling through other mechanisms, such as altered mRNA regulation or stability. It is also possible that these SNPs are not the functional polymorphisms but are linked to a functional polymorphism elsewhere in the gene. These two SNPs are in partial linkage disequilibrium (χ^2^ = 31.4656, p<0.001, d' = 0.751 and r = 0.690). The rs4603829 SNP has not been previously associated with a phenotype while the rs4522666 SNP has been associated with cigarette smoking among schizophrenics [47].

The minor allele of rs4522666 is associated with a higher HA score (Table 4) and the minor allele of rs4603829 is associated with a lower HA score (Table 5). Specifically the rs4522666 genotypes AA and AG are associated with low scores, while the GG genotype is associated with high scores. The rs4603829 genotypes TT and CT are associated with low scores while the CC genotype is associated with high scores. A few individuals had genotypes that gave conflicting predictions of their HA score; specifically individuals that are CT at the rs4603829 locus while being GG at rs4522666 locus. If these individuals are removed from the analysis, the FDR p-values become more robust (two individuals with incomplete data were also eliminated, for a total of 7 persons removed from the analysis). The elimination of these 7 individuals lowered the FDR adjusted p-value for rs4522666 (F _1, 52_ = 17.75, p = 0.0147) and for rs4603829 (F _1, 55_ = 17.27, p = 0.00885). These two SNPs were not significantly associated with any other phenotypes.

**Table 4:** Harm Avoidance Scores by *CHRNA4* rs4522666 SNP.

Genotype	Mean	Std. Error	Count
C/C:	0.568	0.042	10
C/T:	0.404	0.023	29
T/T:	0.393	0.027	25

**Table 5:** Harm Avoidance Scores by *CHRNA4* rs4603829 SNP.

Genotype	Mean	Std. Error	Count
G/G:	0.561	0.037	10
A/G:	0.431	0.024	30
A/A:	0.373	0.027	21
Unknown:	0.291	0.087	3

### Associations with Other Polymorphisms

Our study was small for a genetic association study. The power of our tests to identify large standardized effects (Cohen's *d* = 0.80) with α = 0.05 and a Bonferroni adjustment for 84 polymorphisms (0.05/84 = 0.000595) is 0.43 given our sample size. Realizing this limitation, however, we report on several polymorphisms that showed significant unadjusted associations with one or more of the phenotypes we measured. These associations are listed in Table 6 and include SNPs in *COMT*, *DAT*, *DISC1*, *DRD2*, *DRD3*, *ESRa*, *NET* and *TPH* and a VNTR in *MAOA*.

**Table 6:** SNP Associations with Uncorrected P-values ≤ 0.05.

Marker	P-Value	F-Stat	d.f.	FDR*	Minor Allele Frequency
*Novelty Seeking - Recessive*					
* COMT* rs4646312	0.013	6.484	62	1.000	0.484
* COMT* rs165722	0.016	6.180	60	0.660	0.371
* COMT* rs4818	0.025	5.244	63	0.711	0.492
* NET 2* rs2242446	0.026	5.214	57	0.549	0.246
* DISC1* rs3738401	0.030	4.915	63	0.508	0.300
* DRD3* rs963468	0.043	4.265	63	0.602	0.292
* MAOA VNTR*	0.044	4.256	59	0.522	0.361
* COMT* rs4633	0.049	4.035	63	0.516	0.346
*Novelty Seeking – Dominant*					
* DAT* rs27072	0.001	11.033	63	0.137	0.154
* ESRa* rs3798577	0.021	5.574	63	0.981	0.500
*Harm Avoidance - Recessive*					
* DRD2* rs11214608	0.018	5.894	62	0.507	0.430
* NET* rs40434	0.038	4.488	63	0.800	0.385
* ESRa* rs2228480	0.044	4.206	63	0.622	0.223
*% Safe Gambles Large – Dominant*					
* VMAT2* 2 rs363333	0.014	6.316	64	1.000	0.189
* VMAT2* 5 rs1860404	0.017	5.953	64	0.804	0.212
* NET* 2 rs2242446	0.026	5.243	59	0.786	0.238
* DRD3* rs226082	0.033	4.755	65	0.755	0.299
* NET* 3081	0.048	4.086	63	0.728	0.238
*% Safe Gambles Gains – Recessive*					
* ESRa* rs3798577	0.036	4.598	65	1.000	0.493
* TPH2* 2 rs4290270	0.041	4.359	64	1.000	0.386
* ESRa* rs2228480	0.048	4.050	65	1.000	0.224
*% Safe Gambles Gains – Dominant*					
* VMAT2* 2 rs363333	0.006	8.165	64	0.529	0.189
* VMAT2* 5 rs1860404	0.036	4.578	64	1.000	0.212

* p-value adjusted for False Discover Rate associated with multiple comparisons.

The association between a *DAT* SNP (rs27072) and novelty seeking features an uncorrected p-value = 0.001 and a FDR corrected p-value = 0.137. NS was associated with 4 different *COMT* SNPs, including rs4646312, rs165722, rs4818 and rs4633. With regard to the economic risk attitude measures, two *VMAT2* SNPs (rs363333 and rs1860404) were associated with both SGL and SGG. *ESRa* rs3798577 was the only SNP associated with risk attitudes from both economic and psychology literature.

## Discussion

The results reveal a significant association between a psychological risk attitude measurement and two SNPs in *CHRNA4*. Each of these associations survives correction for multiple comparison testing. There are several reasons why the significant association between HA score and two SNPs in *CHRNA4* is of interest. Neuronal nicotinic cholinergic receptors, including *CHRNA4*, are of general interest because they modulate the release of several neurotransmitters, including dopamine, serotonin, gamma-amino butyric acid (*GABA*) and glutamate in the ventral tegmental area [48]. *CHRNA4* is highly expressed in the central nervous system and is, in particular, important in modulation of mesolimbic dopamine function [49], which suggests it is an appropriate target for studies concerning reward processing and risk attitudes [49].

Furthermore, polymorphisms in *CHRNA4* have been associated previously with response inhibition as measured using cognitive tests such as the Stroop Test, Matching Familiar Figures Test, Tower of London Test and the Continuous Performance Test [50] and as measured by success in smoking cessation programs [49]. Harm Avoidance is also associated with inhibitory response; respondents scoring low on HA have been characterized as having underdeveloped inhibitory responses [51]. Inability to inhibit response is a classic issue with impulse control disorders such as pathological gambling (PG) and previous work has revealed significant associations between HA and PG [4], [5], [6]. These results may also hold relevance for understanding the incidence of impulse control disorders in Parkinson's patients treated with dopamine agonists [10] in light of the role that CHRNA4 plays in dopamine regulation and in light of the data suggesting pre-morbid risk taking behavior is associated with the risk in developing impulse control disorders with treatment of Parkinson's [52], as well as the prevalence of tobacco use among PD populations. Research by Takeuchi et al. [53] suggests that nicotine receptor stimulation can protect against DA neuron degeneration in Parkinson's patients.

The SNPs that provided significant association with HA scores in the present analysis were not the same as the SNPs found to be significant in either Rigbi et al. [50] or Hutchison et al.[49]. One of the SNPs from Hutchison et al. (rs2236196) was located in the 3′ region of the gene, as were both the SNPs found to be significantly associated with HA scores in the present work. We note that the SNPs found significant by Rigbi et al. [50] and Hutchison et al. [49] are also included in the present set of analyses but failed to produce significant associations with Harm Avoidance or any other phenotype. The relevance of these findings can be resolved only after the underlying molecular mechanisms are known. We have preliminary evidence, measuring allelic mRNA expression in human autopsy brain tissues, showing that the 3′ region of *CHRNA4* harbors a functional polymorphism that affects mRNA expression or splicing (W. Sadee, unpublished results); if confirmed, this would further support a possible role for marker SNPs in this region.

The association between the *DAT* SNP rs27072 and NS, which did not survive significance after correction for multiple comparisons (FDR p-value = 0.137), also has a linkage to past research. This SNP has been associated with early onset smoking among Chinese subjects with severe nicotine dependence [54], alcohol withdrawal seizures among a sample of alcoholics [55] and inattention and hyperactivity/impulsivity in a sample of Canadian children [56]. Furthermore, Voon et al. [52] found NS scores help predict the onset of impulse control disorders among Parkinsons patients treated with dopamine agonists. NS was originally viewed as a trait influenced by variation in the dopamine system, with studies associating NS with SNPs affecting dopamine receptors, most notably *DRD4* [20], and others have found associations between NS and *DAT* SNPs [57], but others have also found associations between NS and *DAT* neural density [58].

The significant association between economic risk attitudes and *VMAT2* SNPs is novel in the literature, though we must caution that the statistical significance of this finding does not survive adjustment for multiple comparisons. The rs363333 SNP has been previously implicated in alcohol dependence [59] though our search of the literature revealed no previous behavioral associations for the rs1860404 SNP. Previous research involving *VMAT2* and inherent genetic variation has indicated the gene as a region of interest with respect to risk attitudes. For example, cocaine users lose *VMAT2* protein compared to non-cocaine users [60]. This is of interest as substance addicted individuals have poorer ability to control impulses toward high risk choices in the Iowa Gambling Task [61], [62]. Other work also links genetic variation in *VMAT2* to impulse control issues. For example, Lin et al. [63] find haplotypes within *VMAT2* to be associated with a protective factor against alcoholism while Glatt et al. [64] find haplotypes within *VMAT2* to be protective against PD for women.

Several SNPs in *COMT* were associated with NS, including the rs4818 SNP that Roussos et al. [30] found to be significantly associated with behavior in the Iowa Gambling Task. Other genetic variations in *COMT*, most notably the Val/Met 158 polymorphism, has been associated with sensation seeking among women [65], novelty seeking and reward dependence in Chinese women [66] and extroversion and novelty seeking [25]. Previous research has associated novelty seeking personality traits with PG outcomes [4], [6].

Given the small sample size involved in this study, independent replication of these results with larger sample sizes is necessary to further refine the genetic basis for risk attitude measures. Our exploratory results suggest that employing several risk attitude measures drawn from distinct disciplinary literatures can be important to refining the understanding of any genetic basis as the degree of correlation found between economic and psychological measures of risk attitude in this and other samples tends to be low. Furthermore, while most research on the genetic basis of risk attitudes have focused directly on dopamine receptors and the dopamine and serotonin transporter genes, we find that genetic variation in a broad array of genes with ties to the regulation of dopamine and serotonin might be important for understanding individual risk attitudes. For future directions for genetic studies, the present results set the stage for estimating the cohort size needed to address and replicate the associations identified here. Moreover, consideration of the interaction between the various risk genes, each individually with some potential impact on risk behavior, has the potential to reveal more accurately the impact of genetic factors on risk taking behavior.
